# Extra Supplement.—The Nursing Mirror

**Published:** 1894-01-06

**Authors:** 


### "The Hospital" Nursing Mirror.

Being the Extra Nursing Supplement of "The Hospital" Newspaper.

[Contributions for this Supplement should be addressed to the Editor, The Hospital, 428, Strand, London, W.C., and should have the word "Nursing" plainly written in left-hand top corner of the envelope.]

### News from the Nursing World.

### A CRIMEAN HEROINE.

The name of Miss Harriet A. Tebbutt, whose death was recently announced, must be familiar to many of our readers. She was Superintendent of the General Hospital at Scutari, where she joined Miss Nightingale at the time of the Crimean War. Miss Tebbutt also did excellent service at the Cholera Hospital, Liverpool, where she was assisted by Miss Merryweather, and at different times she took temporary charge of the nursing department at the Nottingham and Birmingham hospitals. Miss Tebbutt has done valuable work in London during the last twenty years, and she will be missed by a very large circle of friends.

### A REASONABLE REQUEST.

The Hampstead Nursing Association certainly deserves the support for which it pleads. It maintains fully-trained nurses for the exclusive service of the sick poor in their own homes, therefore it is hardly likely that the prosperous inhabitants of the neighbourhood will allow the Association to suffer long from its present want of funds for working expenses.

### FULL AND EMPTY WARDS.

"Why do we put up extra beds?" responded the nurse, "I should think that question is well answered by the look of those who occupy them." And following her glances even a stranger can see that unless these couches had been requisitioned, more than one sufferer would probably have died by the way. With this imperative demand for beds for many urgent cases in all poor neighbourhoods, the unoccupied wards at the Metropolitan Hospital, Kingsland Road, are surely amongst the saddest of sights. Fine rooms wasted, because funds are not forthcoming either to furnish them or to maintain the patients for whom they were built. There are large numbers of out-patients, and amongst them bread-winners and poor mothers, who sorely need treatment and skilled nursing, but they cannot possibly get it, the admissions being strictly limited, although the empty wards are silent witnesses to the incongruity to which permits them.

### DESERVING OF SUPPORT.

There is a very useful but not much talked-of establishment at Reigate, which bears the title of "The Brabazon Home of Comfort." In it are received girls and young women suffering from incurable or chronic disease. The only qualifications needed are easily discovered—namely, poverty, and a good character! There are so many young women without homes, and with practically no friends in a position to help them. When the cases are unsuitable for hospital treatment, or the patients are discharged as incurable, the outlook would, indeed, be a sad one were it not for the Home of Comfort, and similar havens of refuge. Members of the Girls' Friendly Society are admitted for eight shillings a week, and non-members for ten shillings. They have skilled nursing and loving care amidst home-like surroundings. It is to be hoped that the increase of subscribers, for which an appeal is now being made, will be cordially responded to. Such admirable work should be greatly extended.

### A GOOD WORKER.

Miss Catherine Newman, the first warden of the Cheltenham Ladies' College Settlement at Bethnal Green, died in December, after a few days' illness. She was trained at King's College Hospital, and afterwards did most useful work in organizing district nursing at Cheltenham. Miss Newman has been greatly valued at Bethnal Green, and a large number of the poor people to whom she had ministered attended the memorial service held at St. John's, Bethnal Green, where an address by Mr. Ingram was received most attentively.

### VALUED SERVICES.

For the last three years a trained nurse has been provided for the sick poor of Broughty Ferry. Her services have been highly valued during this time, and the subscriptions to the Nursing Fund have been on the whole most satisfactory. All those present at the recent meeting of subscribers appeared to agree with Dr. Wemyss when he said he "hoped the institution would become one of the permanent agencies of Broughty Ferry." The chairman asked for liberal support to enable the committee to meet the necessary expenditure.

### A PROMISED HOSPITAL.

At the public meeting called by the executors of the late Mr. Ganderton, at Pershore, it was decided to establish a nursing home in connectien with the proposed cottage hospital. Donations to the amount of £500 have been promised, and this secures to the town an equal sum under the will of Mr. Ganderton. It was remarked at the meeting that the work done in the town by the nurse during the last four or five years had caused all classes of people to set a very high value on skilled nursing. This is certainly most pleasant voluntary testimony to useful work well performed.

### WORTHY OF IMITATION.

The new Nurses' Home at Leeds is to be constructed on the block principle, and will contain forty separate rooms for the accommodation of the nursing staff of the Workhouse Infirmary. The Guardians have wisely decided to make proper provision for the comfort of the trained nurses and probationers they require to attend on their sick poor. The difficulty at present existing with regard to a sufficient supply of competent nurses for poor-law infirmaries will be considerably lessened when other boards follow the good example set by the Leeds Guardians.

### QUEEN'S NURSE AT INVERNESS.

The first meeting of the Inverness Branch of the Queen Victoria Jubilee Institute was recently held in the Town Hall, and much enthusiasm was shown. Eloquent speeches by Dr. Norman Macleod and others ought to secure the liberal subscriptions pleaded for. The services of Nurse Nicolson are highly valued in the neighbourhood, and it is to be hoped that the committee will see their way to maintain a second nurse in the course of another year.

### PROGRESS IN DUBLIN.

There is a scheme under discussion in Dublin for the advancement of the education of probationers by means of courses of lectures, open to members of the nursing staffs of all the hospitals. Such a plan has much to recommend it, if efficiently arranged and carefully thought out, as doubtless it will be.

### AN IDEAL RECEPTION.

"Did the Queen send you herself?" asked one of the poor sick folks at Labrador the other day when a nurse from the Deep Sea Mission finished dressing a wound. Being told that though Her Majesty did not actually send out the doctors and nurses, yet they had her cordial sympathy and approval. "Well, that's all right," said the patient, contentedly; "I guess you'll find your good Queen waiting down at the harbour when your ship comes in!" And no words of the missionary workers could shake the faith of the settler that such would be the case. Her father had been an English subject, and had told her all about Queen Victoria, and she "guessed she ought to know as well as other people" what was to be expected.

### NURSING IN CEYLON.

The subject of nurses seems to be claiming a good deal of attention in Ceylon just now, and doubtless it is well that the adequate training of suitable natives for this work should be thoroughly considered. It is, however, somewhat amusing to learn that the training for service in the Women's Hospital has been suggested as included amongst the duties of the lady doctor! The idea of doctors of either sex being expected, even if competent, to give thorough instruction in the art of skilled nursing, had better at once be abolished. The present race of practitioners is too well accustomed to receiving assistance from intelligent, thoroughly trained women to be likely to consent to any "cases" being left to persons whose nursing experience is limited to the general hints which a qualified doctor may casually bestow. No hospital in Ceylon, or anywhere else, can be properly managed unless a fully-trained, experienced nurse is placed at the head of the nursing department. She must also have power to select and to educate women showing special aptitude for the work.

### HER OWN WISH.

The kindly feeling with which our nurse readers regard The Hospital is often brought to our notice in most gratifying ways. Recently a very touching instance occurred, for Nurse Hosking, who died on 31st ultimo, at 14, Morral Place, Penzance, left special directions that an announcement should appear in our columns as soon as she had passed away. Nurse Hosking was trained for three years in the Manchester Royal Infirmary, afterwards Head Nurse at Hartford General Infirmary for fourteen months, and Head Nurse at the Liverpool Home for Incurables for two years. For the last two years of her life Nurse Hosking worked in connection with the Queen's Jubilee Institute.

### ONLY THREE POUNDS

"Time is money," says the old saw, but apparently it is a much less valued possession at Luton, where the Town Council spent four hours over one meeting last month. According to the reports, a great part of this time was devoted to an argument respecting the sum of three pounds. A young man who supports his mother had the misfortune to contract typhoid, and moreover, he became too ill to allow of his removal to the Corporation's hospital, and pending a dilatory correspondence between various committees, "trained nursing" was supplied to the patient. It is fortunate that the engagement of a nurse was not postponed until the voice of authority had decided who should pay her, as the case was urgent, and the matter after all was not settled at this lengthy meeting. A discouraging statement was made by the Deputy Town Clerk that "any outlay incurred was repayable by the parties to whom the attendance was given." It transpired that the cottage set apart for infectious diseases by the Corporation does not admit typhoid, and the urgency of this disease hardly seems understood at Luton, otherwise there would be some abolishment of red tape in favour of common sense promptitude. The sanitary inspector stopped the poor people with whom the patient lodged from continuing their trade of greengrocers; but the necessity of this step is not exactly apparent, nor is it explained from whom the parties are entitled to compensation for their temporary losses. Amongst the doubts which surround this sum of three pounds sterling, one fact stands out clearly, namely, that all readers must pity the case of any typhoid patient with limited means residing in the borough of Luton.

### SHORT ITEMS.

Nurse Little is doing excellent work for the St. Ives Nursing Association, which was formed last year. —The Lichfield Nursing Institution issues a good annual report. The nurse's work is supplemented in an excellent fashion by the invalids' kitchen.—The committee of the Queen Victoria Nursing Institution at Wolverhampton have purchased a site for a nurses' home.—A fund for providing Christmas comforts for the inmates of the Austin Hospital for Incurables at Melbourne was raised by means of a dramatic reading. This was given in a large ball-room at Croton Hurst, and was under the direction of Rev. John Reid, secretary of the Shakespeare Society.—The Nurses' Institute, St. Peter's, Bedfora, which has been considerably enlarged, will be reoccupied in March next. It will be formally opened by her Grace the Duchess of Bedford. *Nursing Notes* for January contains an excellent summary of the chief events which have made 1893 interesting to nurses.—The Kettering Nursing Association has done some excellent work during the last year, an outbreak of typhoid fever having made a special demand on the services of the nurses.—The excellently-printed quarterly journal, the *Pioneer of Fashion*, has an article in the last number which will interest many old London Hospital workers.

### District Nursing.

### I.—INTRODUCTORY.

NURSING in all its aspects has been so prominently before the public of late years that there are very few persons who have not some idea of what district nursing means. Briefly stated, it is nursing the sick poor in their own homes.

Since her Majesty devoted the bulk of the Jubilee offering of the women of England to extending this work, greater attention has been attracted to it, and it is now generally recognised as a definite branch of the nursing profession. It is work that has existed for long, and has been carried on under various conditions. New systems have been organised to improve its standard and extend its operations. Those interested in its history will find information in the "History and Progress of District Nursing," by Mr. W. Rathbone.

### DIFFICULTIES OF THE WORK.

This work of nursing the people in their homes is much more difficult than is generally supposed, and involves wider and deeper questions than most nurses who propose to enter on it take into consideration. It should be understood at the beginning that there is no sentimentality about district nursing. Nurses who take it up with such an idea are not only disappointed themselves, but do harm to the work. It is really a question of practical economy, the supply of a demand which increases with knowledge of its needs.

### A POWER IN THE LAND.

If the work be continued on right principles it will become a power in the land, and, therefore, its ultimate results, as well as its present effects, must be duly considered. It must be entirely free from all pauperising influences. Free nursing of the poor at home need be no more prejudicial in this respect than is free nursing in hospital, and its moral influence can be made to extend much further, benefiting not only the patients, but their friends and their homes. The power of keeping a home together, when the alternative is the workhouse, is a social question with far-reaching consequences. To shorten the bread-winner's illness by proper attention to the doctor's orders, to save mothers of families from life-long effects of ignorance and neglect, to teach the proper management of infants and children, are gains to the community at large.

### NURSE NOT ALMONER.

The district nurse is a new element in the problem of dealing with poverty and pauperism *en masse*, and her position is hardly yet defined. It has been clouded by the abuse of charity, and to ensure future success the work must be done without suspicion of almsgiving—the hardest lesson for the nurse to learn.

The temptations to give relief are so many—personal friends send money to spend on the cases, charitable people offer to provide whatever is needed, the patients appear so destitute that it seems cruel to refrain. But the nurse must learn to look for the cause of the distress, and let her impressions be guided and corrected by those who study these matters, and whose experience enables them to form a more correct judgment.

Doubtless immediate necessaries for the patient are essential, but whenever possible, the friends should be at some trouble to obtain them rather than the nurse should be called upon to do so. The true way to help the poor is to teach them to help themselves.

### INDEPENDENCE ENCOURAGED.

There is plenty of independence and self-respect among them, though often oddly enough expressed, and the nurse must have a care not to sow seeds of dependence on outside help when sickness and trouble invade the home, which may bear fruit in the workhouse. If the poor are trained to believe relief and nursing go hand in hand, and that everything will be provided for them in illness, what encouragement is there for thrift and laying by for a rainy day? In midwifery work this is even more especially the case—free doctors, free nurses, free clothing bags, free dinners, destroy that maternal instinct of preparation which the very animals possess. It is in this way a nurse can do so much if she only understands its radical importance. Brought face to face with the effects of imprudence by sudden illness, many a man will join a club or benefit society on his recovery.

### BENEFIT SOCIETIES.

By regular payment, when health and wages are good, to the Foresters, the Hearts of Oak, or some other secure society, a member receives regular and sufficient "sick pay" when ill, either in hospital or at home, to keep the wolf from the door, and save the weary anxiety which often retards convalescence out of due proportion to the illness.

Free midwifery attendance should be discouraged except under special circumstances—sixpence a month can be easily spared to secure the maternity bag, though the women should be induced to prepare their own things. A few pence week by week would procure many necessary articles for both mother and child, and in many cases it is only the showing that is required.

### SEEDS OF THRIFT.

The children can be persuaded to save their pennies for the boot club, the Country Holiday Fund, and the Penny Bank, thus sowing seeds of thrift in their young minds. Such domestic details come under the nurse's notice every day, and if only she appreciates their importance, the leaven will spread. And in advocating true thrift she can earnestly protest against the deepest wrong of modern childhood—the insurance of children's lives.

### GOLDEN OPPORTUNITIES.

It is the opportunities given by district nursing that make it so important, and now so many nurses wish to join it, they should realise what the work involves.

It is not nursing alone, though that must be as perfect and well-desciplined as experience and training can ensure, but moral influence, to which there is practically no limit. This is a high and some may consider an exaggerated standard of district nursing, but it is not more than the consequences warrant.

### THE NURSE'S INFLUENCE.

The influence of a good nurse is felt after her services are concluded; her aim should be not only to establish kindly personal relations, but to effect lasting good. It is no easy matter, disappointment and discouragement are ever close at hand. But as the coral reefs of the Pacific are the productions of myriads of tiny workers, so each earnest district nurse who sows the seeds of thrift, self-help, self-restraint, self-respect, in the round of daily work is a helper, however obscure her path of duty, in solving the social problems of the day.

### UNCERTAIN DEFINITION.

Unfortunately, not only do district nurses often fail to grasp what their work includes, but those who desire to aid and extend it are frequently in the same position.

Since district nursing has become more known the demand for nurses is rapidly increasing. The utility of the work is recognised, and those interested in the welfare of the poor feel they are meeting an urgent need by obtaining this benefit for them. At present, however, the popular definition of a district nurse varies with almost every request to supply one.

### TO MAKE HERSELF GENERALLY USEFUL.

Some require a homely, respectable woman to live in the patient's cottage, and who, in addition to attending to the invalid, is to clean the house, prepare the food, and do the washing for the family. Trained nursing experience is not required; three months in a district home is considered amply sufficient. Others ask for a nurse not fully trained, as there is not much sickness in their district, and whose spare time is to be devoted to parochial work, including teaching in the Sunday-school.

Nurses are asked for to attend midwifery and infectious cases, and with few exceptions all inquirers unite in expecting the combination of all nursing virtues for the lowest possible salary.

The Doctors Ignored.

Amongst the many schemes now afloat for introducing nurses wholesale into every part of the United Kingdom there is one point which seems to have escaped notice. Presumably the object is to lessen suffering by providing assistants capable of supplementing the work of the medical men by carrying out orders intelligently, and putting the patients and their surroundings into the most favourable conditions possible. If this be so, is this work being done to the satisfaction of those most interested and most competent to judge, the doctors themselves? No committees, no visiting ladies, however interested in the subject, can decide this point, and yet the judgment of the medical men, who are really most concerned, is not made a prominent feature at all, and too often is altogether overlooked.

Unprofessional Committees.

A nurse is engaged, generally by an unprofessional committee, placed in a district, and doctors and patients are left to make the best of her. There is no standard by which her work can be judged, and no one competent to do it, and so, unless grave faults are committed, she is left to her own sweet will.

The crux of the whole matter lies in the fact that outside the nursing profession, and, indeed, until quite lately, within it, the necessity of more rather than less training for this special work has not been realised.

"Oh, the little more, and how much it is,
And the little less, and what worlds away."

If only those who are interested could be convinced of the fact that disease is no respecter of persons, and therefore the labourer needs the same skilled nursing as the duke, and even more, because he has so much against him. To entrust the nursing of the poor in sickness to women of little or no experience is no more justifiable than to limit their medical relief to unqualified practitioners.

### A Few Remarks on Observation.

By a House Surgeon.

The chief superiority of a trained nurse arises from the fact that she has had special opportunities for noticing the various changes and abnormalities which occur in the course of different diseases, and it is, therefore, important that she should be an accurate observer.

Observation may be taken to mean the mental record of a fact. Many such facts have to be recorded in writing by nurses.

In order to observe well it is necessary that all the senses should actively perform their various functions. In enumerating some of the chief senses, we indicate the kind of knowledge obtained by their use.

*Sight.*—From outline and form we estimate size and quantity. It follows that if observations be made at certain intervals of time, changes of size, of form, or of colour may be accurately noted.

*Touch.*—By muscular sense whether a thing is heavy or light, whether hard or soft, hot or cold.

*Hearing and Smell.*—A clear description of these in words is often difficult until a certain amount of knowledge has been gained, but they are most important for a nurse.

*Taste.*—This is not generally an important part of a nurse's duties, but a knowledge of the taste of drugs is useful.

By means of these senses an accurate mental note of facts or phenomena should be made; then when they occur again they will be promptly recognised. When various facts or phenomena have been recognised classification begins, and instead of being isolated the observations are grouped. These groups having been noted, something more than observation is necessary in order to make them useful. Thus, after certain ones have been isolated, the essential feature of that group has to be recognised or discovered, and this discovery is made by means of intelligence, the most difficult part of education.

We have thus made a rough analysis of the way in which knowledge is gained by the senses. It will be seen that memory is essential, because facts have to be observed at different intervals of time. The chief way in which memory is assisted is by a vivid impression, and this is more easily obtained if a lively interest is taken in the facts or phenomena which come under observation.

### Where to Go.

London Obstetrical Society.—The next examination of candidates for the diploma of midwife will be held on April 11th. Particulars on application to the Secretary, L.O.S., 20, Hanover Square. The next set of classes for preparation for the examination commence on January 12th, at five p.m., at the Midwives' Institute, 12, Buckingham Street, Strand.

Constantinople in London.—Mr. Bolossy Kiralfy is certainly to be congratulated upon his latest and most praiseworthy triumph in the shape of the superb spectacle of "Constantinople at Olympia." It is impossible for anyone who goes there in one visit alone to grasp this colossal exhibition. The play itself is brilliant, the massing of the colours, the grouping, the scenery, and the acting are, taken as a whole, excellent. In spite of a few faults, which time alone can set right—such as the troups not keeping strict step—this great piece is a very pleasing one. The dresses are brought in most effectively, and some of the bands in the triumphal procession give a genuine idea of Oriental magnificence. The introduction of live horses and camels has an especially realistic effect. A striking point at first about the play is that it is dumb, but on such an enormous stage it would be impossible for people in the extremities of the building to hear, and the actions of the performers give the audience a general idea of what is going on. The appearance of acrobats, who are uncommonly good, is quite a novelty, and, after the brilliant colours which precede and follow them, makes a welcome and amusing diversion. The orchestra does its part briskly and with great effect, and takes a leading place in this great and splendid series of pageants. The cigarette manufacturing is extremely interesting, as are many of the wares sold in the mimic shops. All visitors ought certainly to make a point of inspecting in a caique the Hall of a Thousand and One Columns. This hall has been most beautifully arranged, and is approached through a long, dark, awe-inspiring passage, dimly lighted by flickering lamps hung up on the walls. Little points which are remarkable all through Mr. Kiralfy's spectacle make it very life-like, as, for instance, the figures which are placed in the passages leading down to the water in the hall, and the steps leading up from the water to what appear to be doors placed in the wall. We should advise anyone wishing to seek instruction in a most delightful form to go and see, without delay, Mr. Bolossy Kiralfy's "Constantinople in London."

### Causerie from New England.

A PROPOSED COTTAGE HOSPITAL.

MR. GEORGE BILBRUCK, of Portsmouth, New Hampshire, has signified his intention to give the building fund of the proposed cottage hospital the sum of $10,000, without conditions, to be available January 1st, 1894. The trustees already hold a fund of $18,000, and Mr. Bilbruck's gift will make good the amount of $50,000 which Hon. Frank Jones promised when other citizens should have contributed $20,000. This plan of provisional giving is one of the most stimulating and rarely fails. It is expected that work on the new hospital will begin early in the spring. A seaport like Portsmouth, even where the amount of shipping entries is not large, specially needs the convenience of a hospital as an aid to public health.

PROFESSOR BRONSON.

Dr. Henry Bronson died November 26th at his home in New Haven, Connecticut. He had nearly reached his ninetieth birthday, and had been a confirmed invalid for a quarter of a century. He was formerly a professor in the Medical School of Yale University, to which he has given $83,000. His fortune is estimated at a million dollars, and he bequeathed $20,000 to the New Haven Hospital and $10,000 to the Hospital of Waterbury, Conn., his native town.

A CREMATORIUM AT BOSTON.

The first building in the State devoted to cremating the dead is now in process of erection by the Massachusetts Cremation Society. It is situated near the beautiful cemetery of Forest Hills in the suburbs of Boston. The building is of stone, and makes some architectural pretensions to beauty. A chapel for funeral services will be a part of its design. The retort will be ready for use the first of the coming year, and the first body reduced to ashes will be that of the late Lucy Stone, that remarkable woman who verifies Emerson's dictum, "Let the single man stand on his own instincts, and the great world will at last come round to him." In this instance it was a woman who dared to "stand." She lived to see the anti-slavery movement and suffrage for women changed from obloquy to respect. The Cremation Society is in a flourishing condition, and has recently increased its capital stock to $50,000. The idea of cremating is somewhat popular in Boston. But chemists and bacteriologists are throwing upon each other the burden of demonstrating that cremation is a hygienic necessity.

A VICTIM TO LEPROSY.

The heroic element in the character of nurses often finds expression, but seldom in a more marked manner than in the case of Miss Mary Reed. This American nurse, who has devoted herself to nursing among the lepers of India, has contracted the loathsome disease. But in a recent letter to her friends at home, instead of bewailing her hard fate, she asks that fifty dollars of her small salary be withheld and given to the sufferers from the floods in the Ohio Valley, where she formerly lived. Surely in her dwells the stuff of which martyrs are made.

BOARD OF HEALTH.

Every year sees an increased efficiency of the Massachusetts State Board of Health. Twenty-four years ago it began its work in a small way, gradually enlarging its scope, not only by actively assuming control of sanitary conditions, but by stimulating proper legislation and educating public opinion. Cases of illness, causes of death, a record of sanitary needs and improvements are regularly reported at the State House.

ADULTERATION OF MILK.

The Board gives an interesting *résumé* of its inspection of drugs and food, including over 3,000 tests of milk, of which 1,514 were found adulterated or below the standard. Examinations of cattle for slaughter and for export, and the sanitary control of dairies and cow stables are parts of the important work recently perfected. Attention has also been given to the ice supply. It is gratifying to know that manufactured ice was found to contain very few bacteria, the impurities being least when distilled water is used.

PROFESSOR SEDGEWICK'S INVESTIGATIONS.

The State Board is very fortunate in having as its biologist Professor W. P. Sedgewick, of the Massachusetts Institute of Technology. The report includes his original bacteriological investigations on epidemics of typhoid fever in Lowell, Lawrence, Newbury Port, and other cities in the Merrimac Valley; an epidemic of the same disease in Springfield, apparently due to infected milk, and a parallel experience in Somerville, a large suburb of Boston. But the typhoid epidemics in the towns of Cape Cod were attributed to secondary infection from one person to another.

The condemnation of polluted wells, the employment of an officer to enforce the absence from school of infected children in order to suppress an epidemic of diphtheria in Waltham, and prohibiting the dangerous disposal of garbage, indicate the details of the work of the Board. The fact that cities have appealed to these health officers to abate local outbreaks of disease show recognition of their value.

BOSTON DISINFECTING STATION.

A well-equipped disinfecting station in Boston, so long talked of, is at last in working order. Three small outbreaks of typhus fever from immigrants were promptly controlled. The Board urges the imperative need of district sanitary inspectors, hospitals for infectious diseases, and more thorough vaccination. The latter is specially important just at present, when Boston is threatened with an epidemic of small-pox. There are eleven cases at its fine small-pox hospital, a larger number than any time for the past ten years. These cases are causing much anxiety, as they have appeared in different sections of the city, and it has been possible to trace no connection between them.

For the year 1892 Boston had the lowest mortality from typhoid fever of any large American city. One of the questions now agitated is the advisability of having a law regulating the compensation to men detained from their daily labour, in order to completely quarantine infected families.

A HOSPITAL SECURED.

Titchburg is happy in the prospect of a new hospital. This Massachusetts city has over 27,000 inhabitants, and the only accommodation for the sick consists of a few rooms in an almshouse. These rooms cannot be isolated from the rest of the house and have no sanitary conveniences or anything else to recommend them. The late Gardner S. Burbank felt this to be a reproach to the city and bequeathed his entire property, about 400,000 dols., to build a free city hospital after the death of his wife. But the widow desires to see the work begun at once, and the trustees of the will have secured the consent of the legislature to anticipate the legacy and purchased an estate. The house is in prime condition, having been used as the summer house of a wealthy gentleman, and the 400 acres of land adjoining allow plenty of air space for all further enlargement without encroaching on the fine park. The hospital will be ready for occupancy early in 1894.

### Presentations.

THE nursing staff of the Blackheath and Richmond Institution presented a beautiful claret jug to Mrs. L. Smith and Miss Duncan.

THE nurses of the Kent and Canterbury Institute presented Miss Snow, the Lady Superintendent, with a handsome polished oak bookcase.

BEDFORDSHIRE INSTITUTE OF TRAINED NURSES, ST. PETER'S, BEDFORD.—The nursing staff of this institution gave a handsome silver teapot and cream jug with a pretty fancy flower pot to their Honorary Lady Superintendent, Mrs. Ramson, on Christmas Day.

ON December 28th, the nurses of St. George's-in-the-East Infirmary invited the Matron, Miss Wesley, to an "At Home," and in the course of the evening presented her with a beautiful silver cream jug, sugar bowl and tongs, and six teaspoons in a case. A lovely bouquet and a letter signed by all the nurses accompanied the other gifts.

### "First Aid in Accident Cases." Programme of the Argentine Society

1st.—Preliminaries, and object of instruction, aims of the Society, &c.—A general sketch of the structure and functions of the human body—A brief description of the bones, muscles, arteries, and veins—The functions of circulation, respiration, and the nervous system—Esmarch's triangular bandage, and its uses.

2nd.—General direction of the principal arteries, showing the points where circulation may be stopped by digital pressure, by the use of screws, or by Esmarch's elastic bandage—The difference between arterial, veinous, or capillary hemorrhages, the different methods for stopping them.

3rd.—Symptoms of bone fractures, and the first aid to be given in accidents of that nature—The use of splints or other fixed apparatus—Treatment of bruises, sprains, and dislocations—Use of the triangular bandage.

4th.—The first aid to those suffering from shock, resulting from divers accidents; to those who have been stunned, to the apoplectic, inebriate, epileptic, in cases of fainting, and to those bitten by rabid animals, &c.—Immediate treatment of the half-drowned or half-suffocated—Artificial respiration by the Silvester method—Treatment of burns by fire or by boiling water.

5th.—To improvise the means of easing and transporting the sick, or victims of accidents—The mode of employing litters—Transport of the sick by rail or vehicles.

Note.—Examination papers are received at the Public Assistance of Buenos Ayres (Esmeralda 66), where the Argentine Society of "First Aid in Cases of Accidents" is installed; and those who wish to obtain instruction are invited to attend the lectures given on Saturdays from eight to nine in the School for Male and Female Nurses.

Statutes of the Argentine "Society of First Aid."

I.—The Society of "First Aid" proposes to spread the knowledge of the first aid to be given in accident cases—to the medical laity who desire to help their fellow-creatures on such occasions—by founding schools of "First Aid," in which all the first assistance to be rendered before the arrival of the doctor will be taught, registering in the Society all such medical men and students of our faculty who wish to lend their services gratuitously in these cases of accidents, and who will give voluntary aid in the teaching.

II.—The Society is constituted for the registration of all, well-known doctors being considered as honorary members. All doctors and medical students have the right of entry, and those who are neither can acquire it by proving their fitness for examination, or by belonging to some other society of a similar nature. The person entering will be credited with the numbered medal delivered to him, which must be kept ready to hand at all times to permit recognition as a member of the Society whenever it may be necessary. Those examined will receive besides a certificate of competency, with a number corresponding to the medal.

III.—Every member of the Society giving first aid in a case of accident, must make known the number of his medal to the doctor on his arrival, or on the arrival of the patient at the place he may be transported to, so as to relieve himself of his responsibility.

IV.—Instruction will be given principally to those who, by their professions or occupations, are most likely to be present in cases of accidents, and to render aid in the first moment, as, for instance, nurses, watchmen (patrols), firemen, soldiers, sailors, professors, &c. Besides these, all persons may receive instruction who wish to be useful to their fellow creatures in cases of this kind. The Society undertakes to distribute books, parallelograms, models, bandages, &c., for the purposes of teaching, for which object it will be necessary to collect funds by donations, entrance fees to the lectures, &c.

V.—The examinations will take place in March, June, September, and December before a committee of medical men, and will be essentially practical, giving the reason for each thing according to the method of the five conferences of Esmarch.

VI.—The registration book will for the present be under the superintendence of the Directors of the School for Male and Female Nurses of the Public Assistance of Buenos Ayres, who will have to record the name, number, age, nationality, present occupation, date of entrance, and examination made —in the latter case with the signature of the examiners.

### Novelties for Nurses.

### KNITTED RESPIRATORS.

Half a dozen knitted respirators were forwarded to us for distribution with our Christmas parcels, and we sent them to the North London Hospital for Consumption. They cost but 2d. each to make, and can be burnt when done with. Any one wishing for the pattern can obtain it, and directions for making, by sending 3d. to Miss Helen Grafton, Heysham Hall, Lancaster. The proceeds are sent to the Indian Hospital at Lytton, New Westminster.

### A SICK-ROOM THERMOMETER.

Messrs. Garrould, of Edgware Road, who are making a speciality of nurses' requirements, are now selling a most useful thermometer for 6d. At such a price this thermometer should be in great demand. No nurse will hesitate in requesting the essential thermometer when nursing private cases now one so inexpensive can be procured. We have tested the specimen sent to us and found it quite reliable.

### Our Examinations.

We did not receive so many answers as usual to our examination question for December, probably because preparations for Christmas engrossed the stray hours which nurses generally devote to their own affairs.

Some answers are good, and all show that the seriousness of influenza is acknowledged, and its demand for immediate and intelligent treatment appears to be thoroughly comprehended. It is satisfactory to see that the idea of keeping the patient quiet, warm, and isolated is generally accepted, and that the need for a doctor and for adhering implicitly to his orders are facts brought prominently forward by most competitors.

Examination Question.

Describe the nursing of a case of influenza, and state what a nurse should specially watch for.

Prize Answer.

In nursing a case of influenza the patient and nurse should be completely isolated from the other members of the household, and in every detail the form of illness should be considered infectious, and treated as such. The temperature of the room must be evenly maintained, and not allowed to fall below 65 deg. Fahr. A fire should be kept constantly burning. The patient's bed-clothing should be warm but light, and flannel should be worn next to the skin. All chills must be strictly guarded against. The patient's temperature must be taken every four hours, oftener if necessary. The nourishment given should be liquid, and cooling drinks included while the temperature remains high. The nurse should carefully note any fresh symptoms that may arise, such as delirium, shivering, cough, expectoration, &c.; watching for any signs of lung mischief, one of the principal complications of influenza being pneumonia. The patient should not be allowed out of bed too soon, otherwise a relapse may set in, and complications are then more likely to occur. The condition of the bowels and kidneys should be noted and reported to the doctor, and his directions implicitly obeyed in the smallest detail. Edith Ashenden.

Question for January.

Describe the nursing of a case of diphtheria, and the special precautions necessary.

Answers must be sent in by January 20th, written only on one side of the paper, accompanied by full name and address, directed "Nursing," care of Editor of The Hospital.

### A Stroll Round the Wards.

BY A WANDERER.

"Do you move the patients just as you like on Christmas Day?" I asked a sister, who handed me a steaming cup of coffee.

"Decidedly not," was her prompt reply. "We get the doctor's sanction about each separate case, and we don't even carry their beds into the adjoining ward for our entertainment without first asking leave. It would never do," and a slight smile flitted over her serious face as she thought probably of "complications" which were too deep for me to fathom. Then she turned to help a weakly girl to adjust her tea-tray, and I wandered on.

An aged woman lay in a bed a little apart, so I lingered to speak, but before I could do so she broke out—

"Eh, then! ye think I'm lonesome?" I nodded.

"Yer wrong then; the nurses they was thinkin' the same, and they asked the doctor for leave to shift my bed up near by the pianner, but they was kinder still after when they said I might just please myself."

"Yes, but grannie, it would amuse you to be with the others."

"No, no; I'm weary, and I cannot bear to be moved. I'm thinkin' I've had enough Christmasses on this old earth. I'd be glad to try a change and go a bit higher up," and she sighed again wearily.

"But she's getting better," whispered a nurse, "only she's dull and very poor, and we are going to try and find some of her relations. She lives alone in a miserably poor place."

Again the sad side of things comes uppermost as I stop in another ward where two little lads lie in one cot, nine years old at the most, perhaps they are less.

"No, they b'aint relations," says a man sitting near; "they are just put together for company whilst the singing's goin' on."

"They lie very still and straight," I commented, as I noted the caution with which the little fellows moved as each was challenged to hold out his hand for an orange

"Well, mum," said the man, with a quick glance from me to the boys, "I guess you don't often see hip disease patients—pretty bad ones, too—movin' more than they can help."

I asked no more, but with a quiet "Good day" left the big man, who appeared to forget his own ailments while pitying the sorry cases of his small neighbours.

In a great accident ward cheery voices were joining in the chorus of a popular song, and a little apart was a bed in which lay a strangely motionless figure. But the face was calm and the eyes rested affectionately on the woman beside him. She was his wife, and in her arms lay a month old babe, whilst two dear little lads sat open-eyed with wondering admiration of the gay and novel scene. They had toys and sweets and were enjoying a "very happy Christmas" without doubt. But though the man's calm patience was reflected on his wife's quiet face, there was something more in her expression, and it made me seek for further knowledge of that little family group.

Infinite pity was in the nurse's voice and eyes. "A fractured spine," she said softly, "he's very bad. We asked the family to spend the whole day here. Of course he's on the 'Dangerous List,' poor fellow."

"I shall never forget this Christmas, husband." Did the woman say it or was I fanciful as I stepped further back and left the little party surrounded by an atmosphere of comprehensive love and pity. "A Happy Christmas," said a merry voice, and in the next ward I found a convalescent typhoid patient was the speaker. "I've made a wonderful recovery," said the little maid, uttering the words slowly lest she should not repeat correctly the doctor's words. "'Peace on earth, goodwill towards men,' are facts not hollow utterances in such wards as these," said a parson just behind me.

### Christmas Entertainments.

FOUNTAIN FEVER HOSPITAL.—Residents at a fever hospital inhabit a world of their own, and although it is impossible for the wards to be thronged with outside visitors Christmas is often made pleasant to patients and nurses. In each ward at the Fountain Fever Hospital a fine, heavily-laden Christmas tree was provided, roast beef, plum pudding, cake, oranges, and apples being also supplied to all who were well enough to enjoy them. Fortunately there was an unusual proportion of convalescents, and therefore the festival was seasonably observed. Carols were excellently sung, and a procession of doctors, nurses, convalescent patients, and servants went the tour of the wards, increasing in extent as it passed along. Later in the evening a loud knock was followed by the entrance of Father and Mrs. Christmas, who were admirably represented by two of the staff. Patients confined to bed were not forgotten, for Father Christmas found his way, with an appropriate gift, to each of them.

WEST LONDON HOSPITAL.—The inmates of this hospital had a very pleasant Christmastide. On Christmas Eve each ward was visited by a number of lady choristers, and early on Christmas morn the nurses awakened the invalids by singing a selection of carols. Mrs. Spender and other friends provided a sumptuous tea. Some of the nurses afterwards entertained the patients by performing a May-pole dance in each ward in flower costume, other of the nurses giving a spirited representation of Mrs. Jarley's wax-work show.

BRADFORD INFIRMARY.—The inmates had an excellent dinner provided for them on Christmas Day, according to custom, by the Mayor, and various donations were received of money, clothing, and toys. A goodly company assembled at noon, and the entertainment was appreciated most thoroughly by the patients, who alljoined heartily in thanking the Mayor of Bradford for the good things provided.

DREADNOUGHT SEAMEN'S HOSPITAL, GREENWICH.—A concert was given to the patients of this hospital by Mrs. Frederick Smith on the 30th ult., and the nurses' entertainment took place on January 2nd, when Mr. Nairne, the deputy-chairman, kindly volunteered the services of his band. The Christmas tree for the children will be lighted at four p.m. on the 6th inst

ST. GEORGE'S HOSPITAL.—A Christmas tree covered with toys was provided for the children who spent Christmas in St. George's Hospital. In each ward a special tea was served at five o'clock. Carols were sung by the visitors, and recitations given by the students. Many useful gifts for the patients were also presented by various of the guests.

CHILDREN'S HOSPITAL HOME, KINGSTON HILL.—Christmas Day passed very pleasantly at the hospital home, where the little patients were regaled with turkey and plum pudding, and received many useful gifts from the handsome tree.

ROYAL ALEXANDRA HOSPITAL FOR SICK CHILDREN, BRIGHTON.—The wards of this hospital were prettily decorated, and a very happy Christmas was spent by the children. All who were well enough partook of a famous "high tea." A heavily-laden Christmas tree supplied pretty gifts to the little patients, and a musical entertainment and excellent magic lantern display were also provided.

BROMPTON HOSPITAL FOR CONSUMPTION.—Carols were sung in the hospital chapel on Christmas Eve and in the wards on Christmas Day by the nurses. The patients' dinner consisted of turkeys and plum puddings, and the galleries were prettily decorated with evergreens. In the evening an excellent selection of music was followed by various games, and on Boxing Day the usual distribution of gifts from the Christmas tree took place, a handsome present falling to the share of each patient. A concert and performance of the piece entitled "A Pair of Lunatics" gave general satisfaction.

The London Hospital.—The Christmas tree entertainment at this hospital took place on New Year's Day, and the weather was most favourable for the crowds of visitors who assembled on the occasion. A great many guests assemble at this annual reception, but on Monday last the gathering was unusually large. The whole of the hospital, as well as the nursing home, were visited during the afternoon by a constant stream of people, who seemed immensely interested and gratified by all that they saw and learnt of this vast institution, which was once aptly termed by the Chief Rabbi, in an eloquent speech, "The true People's Palace of the East-end of London."

Essex and Colchester Hospital.—The Christmas entertainment at this hospital was particularly successful this year, and the day passed pleasantly. The dinner consisted of roast beef and plum pudding, and an excellent tea for the patients was provided by two gentlemen on the committee. The decorations were very pretty, and a capital programme of music was carried out. On Boxing Day two farces and some good music filled up a most enjoyable evening.

Abbey Poorhouse Hospital, Paisley.—Through the kindness of a few ladies Miss Holliday, the Superintendent, was enabled to provide an excellent dinner for the patients in the above institution—fruit, sweets, cards, &c., being distributed. The children each received a toy, and in the afternoon tea and cake were served. A concert was afterwards given in one of the large wards, and was well attended by the patients, who thoroughly enjoyed it, many expressing a wish that Christmas would come more than once a year, feeling that even a poorhouse can be made bright and cheerful at that season. Another tea and concert took place on the following Thursday.

The Metropolitan Hospital.—The Beulah Musical and Dramatic Society gave an entertainment on Tuesday evening to the patients at this hospital. The wards were decorated, and a handsome Christmas tree in the children's ward was covered with toys. A present of linen was given to each patient by the North London Needlework Guild.

Huddersfield Infirmary.—The annual Christmas tree and entertainment, provided by a committee of ladies, was held on December 28th in the large accident ward, which was decorated with evergreens and seasonable devices, and every patient able to be present was there. Each patient, nurse, and servant received a useful gift from the Ladies' Committee. On the following evening the annual supper and dance for the residents, nurses, and servants took place, and a most enjoyable evening was spent. The Ladies' Committee also provided the good things for this festivity.

Watford Cottage Hospital.—The wards and corridors of this little hospital were tastefully decorated for Christmas. The dinner of turkey, plum pudding, &c., was provided by friends, and was thoroughly appreciated. A large bran pie was the source of much amusement, the gifts in it being furnished by the matron and nurses. A handsome present had previously been given to each patient by the Honorary Treasurer. On the following Thursday a concert was arranged by friends of the Matron (Miss Atthill), and some clever conjuring tricks and excellent recitations were also part of the pleasant entertainment.

Evelina Hospital for Sick Children.—On a cold, dull day frequenters of Southwark Bridge Road cannot but be struck by the unloveliness of that thoroughfare. Visitors, however, who journeyed down on Wednesday afternoon to the Evelina Hospital, forgot all exterior dreariness when they passed the threshold of the prettily decorated building. The numerous flags and graceful wreaths on walls of wards and corridors were arranged by the firemen belonging to the station next door to the hospital. Their designs were most tasteful, and so were the decorations done by the nurses. But the general condition of cleanliness and order observable throughout the institution were, after all, the best decorations. A fine Christmas tree stood in the centre of each ward, and toys from *Truth* were also much in evidence.

### The Sussex County Hospital.

(Contributed.)

It seemed as if the patients had forgotten all their suffering in the joy of Christmas. Long before the day arrived the nurses' spare time was all taken up with rehearsals for the entertainments. On Christmas Eve pillars were twined with ivy and evergreens; and absorbent wool, and fairy lights and artificial "Jack Frost" abounded. The beds were draped with art muslin or wreaths of greenery, and finished with artistic ribbon bows. Chinese lanterns were strung from one end of the ward to the other; and the nurses, although tired, looked happy, and the sisters did their utmost to make every one at ease. The Matron went round the wards and gave her approval. Gifts for each patient were placed by their sides in their stockings by the night nurses—in fact, not only stockings, but pillow-cases—and boots and trousers were seen suspended from pulleys in the men's wards. "I hope I'll have a good catch," said one, as he fastened a fishing-net to his crutches. Another found his treasures in an irrigator! Scarcely had twelve o'clock struck when a hand was stretched out and the receptacle fingered, and smothered laughter followed. One mother's face was a picture as she exclaimed, "That'll just do for my boy." Then the night sister had to see everything and to share in the general ecstasy to the best of her ability.

Fairy lights transformed the wards, when in the distance the sound of approaching voices was heard. A long procession of nurses and ward maids, nearly fifty in number, headed by the Matron, appeared, and each carried a lighted candle in her hand as she joined heartily in the glorious old carols. From ward to ward the procession passed on, the voices growing faint in the distance. At breakfast each nurse found a useful present on her plate, and at seven o'clock was at work in the wards once more. The doctors went their rounds with tobacco, matches, and pipes for the men. At twelve o'clock the patient's dinner was served, and almost everyone able indulged in roast beef and plum pudding. That over the nurses assembled for their dinner, all but the night nurses dining together, with the Matron and doctors in attendance. The night nurses rose in time for the patients' tea at four o'clock, and after a little rest the series of entertainments began in the wards. These passed off most successfully, and concluded by all assembling in "Vallance" for "Auld Lang Syne," a complete circle being formed. "Let the patients join hands, too," said the House Surgeon, and so from bed to bed hand grasped hand, and the familiar verses were sung by a multitude of happy voices. "Again!" said a doctor; "And again!" shouted another. "Once more for the Matron," and "One more for Sister Vallance," and so on, till every sister was named, and a patient added, "Now, one for the doctors," then "One for the patients" after. Patients were quickly put to bed, the porters having carried some of the little ones to see and hear everything, and helping by their good humour and willingness to let every one have a peep; and "Well! I have enjoyed myself" and such-like expressions were to be heard on every side. Tired out with the day's excitement, pleasure, and surprise they slept, some of the boys carrying the fun of the past day well into the night by wearing the paper caps and masks obtained from the bon-bons.

☞ For Nurses' Looking-Glass, Book Reviews, Everybody's Opinion, and Reading to the Sick, see page cxxxix et seq.

### The Muses Looking Glass.

### WOMEN WRITERS: THEIR WORKS AND WAYS.*

In this first series of biographical sketches Miss Hamilton's object is to "tell the life stories of some famous women writers—how they attained success, and how they enjoyed it—for the benefit of the many who have neither the time nor the opportunity to consult more elaborate works," and in this she has eminently succeeded. Possessing the happy knack of condensing much information into a comparatively small space, the author has brought together these brief biographies in a thoroughly readable and interesting form. This pioneer group of literary women commences with Frances Burney, who, in 1778, published "Evelina," and extends over a period of some fifty years, comprising many names familiar as household words. Amongst them we find those of Mrs. Inchbauld, now best remembered by her "Simple Story," though her literary reputation was made by the success of her plays; Maria Edgeworth; Susan Ferrier, the authoress of "Marriage" and "The Inheritance"; Mary Russell Mitford, and Hannah More. A comparison of the rival claims to permanent distinction amongst these earliest authoresses of the nineteenth century throws light on the curious unreasonableness of fame. Perhaps of the nineteen women writers of varying merit and reputation, whose life stories are here given, Jane Austen was the one who attained the least degree of contemporaneous popularity; yet of all, hers are the books which have gained a place among English classics. Whilst Lady Morgan earned by her pen thousands of pounds, the profits on Jane Austen's published works at the time of her death had not amounted to £700. How little appreciation was given to the inimitable studies of character which fill the pages of her novels is shown by the contemporary criticisms collected through her friends, some of those on "Emma" being so unfavourable that "they could not be reported to the author." The public taste had been vitiated by the stronger meat of such writings as those of Mrs. Radcliffe's and others of the same school, and the delicately minute character painting of Miss Austen was not to become popular all at once. But in a few years widely different views prevail. Sir Walter Scott's opinion, given in his Diary for 1826, is well known: "The big bow-wow strain I can do myself like anyone now going, but the exquisite touch which makes commonplace things and characters interesting is denied me." Lord Macaulay was one of Jane Austen's warmest admirers, and Mr. G. H. Lewis owns he would rather have written "Pride and Prejudice" than any of the Waverley novels

Born in 1775 in the village of Steventon, in Hampshire, of which her father was rector, Jane Austen spent there, in complete retirement, the first twenty-six years of her life, and it was during this uneventful period that her first three novels were written—"Pride and Prejudice," "Sense and Sensibility," and "Northanger Abbey." "Pride and Prejudice" was not published until fifteen years from the date of writing, but was always the one of her works dearest to the heart of the authoress. In a letter to her sister she says: "I want to tell you that I have got my own darling child from London. Miss B. dined with us on the very day of the book's coming, and in the evening we fairly set at it and read half the first volume to her. She was amused, poor soul! That she could not help, you know, but she really does seem to like Elizabeth. I must confess that I think her as delightful a creature as ever appeared in print, and how I shall be able to tolerate those who do not like her, at least, I do not know."

After Mr. Austen's death in 1805, his widow and her two daughters, Jane and her elder sister Cassandra, finally settled at Chawton, in Hampshire, where one of the sons had come into a property. Here Miss Austen wrote her three best novels, "Mansfield Park,' "Emma," and "Persuasion." "After 'Mansfield Park" was published," we are told, "she began to be afraid she had written herself out," but "Emma" admittedly ranks as high as any of her writings, and "Persuasion" certainly shows Miss Austen at her very best. This last was finished in August, 1816, and was not published till after her death. Jane Austen's character was sweet and loveable. During her last illness, which was upon her even while finishing the last chapters of "Persuasion," her thoughts were ever more of others than herself. She died in July, 1817, and was buried in Winchester Cathedral. "She has become a classic. Notwithstanding the ever-flowing tide of new novelists, fresh editions of Jane Austen's works are continually being issued. When young writers ask despairingly what writer they ought to study, every good judge of literature will answer at once—Jane Austen. Her subtle humour, her delicacy of touch, her innate perception of character, exalt her to the rank of genius."

We find amongst many of these early authoresses of the present century a strong tendency towards dramatic writing. The plays of Mrs. Inchbauld, Hannah More, and Joanna Baillie attained much popularity in their day. Joanna Baillie's "Plays on the Passion" drew forth admiring criticism. "Women," says Byron in his Diary, "except Joanna Baillie, cannot write tragedy; they have not seen enough, or felt enough, of life for it." Joanna Baillie was also the authoress of some charming Scotch songs. From her pen came "Wooed and Married and a'" and "The Weary Pund o' Tow." Scotland is rich in more than one poetess whose songs have become national possessions. Lady Nairn (Caroline Oliphant) may be accorded the first place. Her Jacobite songs, breathing in every line that all-powerful chivalrous loyalty which was the peculiar heritage of the Stuarts, will never cease to be popular. Imbued from her earliest youth with passionate devotion to the deposed race, Caroline Oliphant breaks naturally into the inspiring melodies which were sung round the Scottish hearths with loyal fervour. Lady Anne Barnard has immortalised the pathetic story of "Auld Robin Gray" in words unsurpassed for simple pathos. A sequel to this story was afterwards written by the authoress, who was annoyed by "hearing a continuation of 'Auld Robin Gray' sung in the streets," in which, after Auld Robin's death, Jeannie becomes the happy wife of her Jamie:—

> Wi' a bonnie wee bairn, th' auld folks by the fire,
> Oh, now she has a' that her heart can desire.

Miss Hamilton deplores the absence of any good national song or ballad writer among English authoresses. Yet while none have caught the popular fancy like their sisters in Scotland and Ireland, there have not been wanting English poetesses of no mean order. Mrs. Barbauld was one of these. A poem of hers on "Life," written in "extreme old age," was accorded a unique tribute of admiration by Wordsworth. A copy of Mrs. Barbauld's works had been presented to the poet's sister. The concluding lines of this particular poem run as follows:—

> Life! we've been long together,
> Through pleasant and through cloudy weather;
> 'Tis hard to part when friends are dear;
> Perhaps 'twill cause a sigh, a tear;
> Then steal away, give little warning.
> Choose thine own time;
> Say not good night, but in some brighter clime,
> Bid me good morning.

"Long after I gave these works to Miss Wordsworth," says Mr. Crabb Robinson, "her brother said, 'Repeat me that stanza by Mrs. Barbauld.' I did so. He made me repeat it again, and so he learned it by heart. He was at the time walking in his sitting-room at Rydal, with his hands behind him, and I heard him mutter to himself, 'I am not in the habit of grudging people their good things, but I wish I had written these lines—

> Life! we've been long together,
> Through pleasant and through cloudy weather.'"

With the story of the brilliant Lady Blessington, whose writings were celebrated in their day, we come to the end of this series of very interesting biographies, amongst which will be found many other names of equal merit with those we have here briefly touched upon, and for which we must refer our readers to the book itself.

* "Women Writers: Their Works and Ways." First Series. By Catherine J. Hamilton. (Ward, Lock, and Bowden. Price 2s. 6d.)

### The Book World for Women and Nurses.

[We invite Correspondence, Criticism, Enquiries, and Notes on Books likely to interest Women and Nurses. Address, Editor, THE HOSPITAL (Nurses' Book World), 428, Strand, W.C.]

TALES IN VERSE. J. A. GOODCHILD. (London: Horace Cox. 1893.)

Mr. Goodchild commences this small volume of poems with a preface, which we feel is quite uncalled for on the part of the author. Here he tells us his verses are "not for the critic," though, he further adds, "they are not prohibited from review." A review then, we learn for the first time, is not a criticism, but something else. Mr. Goodchild has not quite clearly defined his interpretation of what constitutes a "review" proper, but between the lines we gather it should be something in close analogy with an *éloge* to pass muster in this writer's mind. The right, in his own individual case to this immunity from literary criticism the author bases on the fact that he himself is no "professional" critic, but a "desultory student." Now, in the verses themselves, Mr. Goodchild has shown himself something better than a mere "desultory student." He has written verse for 20 years, and in the present volume of poems there are many attractive lines; an originality of subject matter and of treatment, as in the instance of "Secrets," "In a Studio," and "The Trinity of Thalkove." These three, which we have specialised, show some considerable imaginative power and a certain freshness of thought. The author's strength evidently lies in poetry; he should recognize this, and when the "Tales in Verse" pass into a second edition, he would do well to omit the few prefacing lines of challenge to unseen critics, which, worded as it is at present, does little to increase Mr. Goodchild's literary reputation.

A PRACTICAL HANDBOOK OF MIDWIFERY. By FRANCIS W. NICHOL HAULTAIN, M.D., F.R.C.P.E. (London: The Scientific Press (Limited), 428, Strand, W.C. 1894. Price 6s.)

THIS work is of the nature of a series of demonstrations, and is stated to be intended mainly as "A practical help to the busy practitioner."

It is an eminently practical and really useful addition to the long list of manuals on obstetrics, and it appears to have been well considered before being put into words, the errors in it being those of omission rather than o commission.

It is really a modern book, and, as such, is extremely interesting, the contrast between the books one used as a student and the present one is very marked. One of the points which strikes a reader of this reflection of modern thought and method is the very extended use of the forceps advocated, and in this respect especially the adoption of an axis-traction forceps as the routine practice in preference to the ordinary pair with the double curve. In contrast to this is the advocacy of digital dilatation of the os. Another noticeable point is the very extended use of chloroform recommended; this savours rather of the operating theatre than of the ordinary lying-in chamber, where nowadays the doctor is expected to possess something like a double, if not a treble personality. Any method by which one man could be enabled to give an anæsthetic so as to induce the second stage of anæsthesia without requiring his constant attention would indeed be a boon, but many of the operations which have constantly to be performed, and which are recommended to be done under an anæsthetic in this book, have in practice to be performed without one.

We miss some valuable methods of operation in difficult labour cases; thus in face presentations no mention is made of the very simple but very effectual method of causing rotation in mento-posterior positions by keeping the forehead up and pressing the head round. Where this is done early there is usually no difficulty in these cases.

The use of Champelier de Ribes' bag for dilating the cervix uteri is highly recommended, but no mention is made of Barnes' bags, although Dr. Barnes has stated, we believe, that he tried the same shape prior to deciding on the fiddle pattern.

Having pointed out what appear to us to be the principal defects of the book, we pass on to its merits, which, as already stated, are very considerable. It is, as a matter of fact, a very convenient condensation of the main facts and factors of midwifery. It is of a size which admits of its being readily carried in the obstetric bag, and, if there, will save anxiety about the correctness of one's memory, which is only too apt to be well founded in the early hours of the morning after a hard day's work. There are a number of most useful tables, and the chapter on diseases of the membranes is especially interesting and well put.

The description of the major operations is rather short, but is probably adapted to the length of time available for thinking over the case before operating. There are many points of great practical value about the work, and it is so arranged as to enable the reader to grasp rapidly the main points of the difficulty before him in respect to causation, diagnosis, treatment, and results.

The book would be of still greater value if it were interleaved, and if a list of references to standard works were included.

### Queen Victoria's Jubilee Institute for Nurses.

HER MAJESTY has been graciously pleased to approve the following names being added to the Queen's Roll of the above institute for nursing the sick poor in their own homes:—

ENGLAND.—Superintendent: Ada Jennings, Torquay. Nurses: Elizabeth Macintosh, Woolwich; Ellen Dixon, Portsmouth; Elizabeth Hill, Liverpool; Sarah B. Caldwell, Liverpool; Isabella C. Graham, Liverpool; Maude M. Wray, Liverpool; Elizabeth Shackleford, Liverpool; Elizabeth Gelletly, Liverpool; Margaret Roberts, Liverpool; Elizabeth A. Daniels, East London; Amy Whitaker, Torquay; Margaret Carrick, Torquay; Annie Peterkin, Bridgwater; Annie Lunn, Northampton; Helen B. Walker, Northampton; Maria Bayes, Salford; Mary E. Smith, Salford; Harriet A. Barber, Salford; Edith Knight, Gateshead; Hannah M. Roberts, Bramley; Charlotte S. Cooke, Louth; Hannah Lloyd, Kettering; Bertha Garnham, Ryde; Amy Cooper, Chelsea; Margaret Allison, Ashton-under-Lyne; Sarah L. Barnett, Worcester; Sarah T. Farrell, Kingston; Elizabeth Emuss, Manchester; Annie Worsnop, Garston; Laura L. Watd, Hendon; Rose Russell, Camberwell; Matilda Fitzgerald, Battersea; Annie Lawry, Dover.

RURAL DISTRICT BRANCH.—Ann Ruberry, Warren; Louisa Hill, Whitchurch; Sarah E. Hart, Ide Hill; Alpha H. Fenton, Charlton Kings; Kate M. Taite, Stonehouse; Ellen T. Taylor, Shoreham; Jenny T. Wolfe, Gotherington; Agnes Affleck, Erdington; Florence Sims, Upton-on-Severn; Lucy Haynes, Sherborne.

WALES.—Jane Morris, Beaumaris; Rose Deacon, Bangor Elizabeth Davies, Carnarvon; Mary E. Fletcher, Cardiff; Harriet A. Johnson, Cardiff; Hannah Jones, Denbigh.

IRELAND.—Annie FitzPatrick, Dublin.

SCOTLAND.—Edith M. Halliday, Edinburgh; Mary E. Beaumont, Edinburgh; Rose A. Heath, Dundee; Jane S. Croll, Kirkudbright; Annie Browne, Bothwell; Margaret Dickie, Johnstone; Eliza Myers, Banchory; Mary B. Robertson, Wick; Mary H. Macfarlane, Tobermory.

### Everybody's Opinion.

A CORRECTION.

THE MATRON OF THE INFECTIOUS DISEASES HOSPITAL, MILL ROAD, CAMBRIDGE, writes: Allow me to correct your statement that a nurse recently appointed by the Lowestoft Board of Guardians had been three years at the Cambridge Infectious Hospital. An assistant nurse recently appointed by the Guardians of the Oulton Union, near Lowestoft, had been here between two and three months as temporary assistant nurse.

WORKHOUSE INFIRMARIES.

"ONE WHO KNOWS" is glad that her remarks have brought forth a reply from a nurse who was probably trained at Marylebone Infirmary. It is well known that that workhouse infirmary is not to be classed with other similar institutions. It is, the writer believes, the only one where the Matron is the undoubted head of the female staff, and therein is a great secret of the superior tone throughout. The writer did not quote that infirmary. She named those where a difficulty was found in obtaining a good class of nurses, and the facts stated in the reply add weight to her words. If the same changes are made elsewhere which have been made at Marylebone, there will in future be no difficulty in obtaining suitable nurses for workhouse infirmaries.

THE THREE MONTHS' COURSE.

LADY BAKER, honorary secretary of the Dorset Health Association and original promoter of the three months' technical nursing scholarships now under debate writes:—The writer of the paragraph thus headed in your last issue has evidently formed an opinion upon imperfect premises. The value of a three months' course depends entirely upon what it consists in and for what purpose it is given. To a young inexperienced girl it would be of little use except to test her capabilities and prepare her to work under an older and more experienced woman. To one who had already had a long experience of district nursing under a local practitioner, three months in a hospital would undoubtedly add to the efficiency which he is ready to vouch for—or to a woman accustomed for years to practise satisfactorily as midwife in a country district under the supervision of the medical men, a further special course of training and testing under a hospital-trained and certified London midwife would be as invaluable as lessons from the first masters to a country professional in art. As long as the efficiency of the nurses of whatever grade, from the highest to the lowest, is tested by an unbiassed medical committee and corresponding certificates given after due examination, as in the Dorset Health Association, surely the lady critics may rest satisfied that they will not be allowed to profess to be what they are not. Where, however, the only test of a nurse's efficiency rests either on her own assertion or the decisions of ladies' committees, instead of on professional judgment, the matter may be open to grave doubt, and most unsuitable women may for a time be allowed to assume duties for which they are totally unfitted, though they are scarcely likely to be called in where serious danger arises or allowed to remain by the medical men in charge if other help is forthcoming.

### Suggestions for Australia.

THE regulations concerning outbreaks of cholera framed by the Dresden Sanitary Convention and signed by the principal European powers, were received recently by the Premier of Victoria from the Imperial Government, and are now under the consideration of the Central Board of Health.

### Wants and Workers.

WILL anyone kindly give an old cot or cots, or small bedsteads, for the use of the children in the Milicent Home, and any old blankets, linen, or rugs? Address Matron, Milicent Home, Sandown.

### For Reading to the Sick.

"A CALL."

WE are laid by for a few weeks with an illness which promises to pass away without serious results. It has been sharp for the time, making our friends look grave and anxious and our medical attendant peremptory that his orders should be strictly obeyed; but there has been no immediate danger, and we are now turning the corner to convalescence. We shall have some time yet to lie still or creep about for air and exercise, so we may as well think over the past and on the future.

Personally, we have hitherto been too much engrossed with the passing moments, with our aches and pains, our changes from hour to hour; we have felt very bad, and too much engrossed by our discomfort to ponder on our death. If we have cast our thoughts that way it has been to comfort ourselves that our youth or our good constitution would surely pull us through in the end, and to hope the moment will soon arrive when we shall be well and able again to enjoy the pleasures and join in the thousand and one schemes and employments which accompany good health.

Now we know there is nothing so desirable for an invalid as to be hopeful. Competent authority, too, tells us that nothing retards or helps on recovery as a cheerful spirit does. "The merry heart doeth good like a medicine," yet it is equally true that it is well for every illness to be made an occasion for serious thought and self-examination. Why, we may ask ourselves, has this attack of rheumatism, or bronchitis, or a broken arm, or sprained ankle been sent us? We have been living good sort of lives, not doing more harm to our neighbours than other people. Possibly that is just why our sickness has come, because we make our neighbour's opinion our rule of life, not God's laws. But He loves His children so well that He stops us on our road and calls us out of the heat and tumult of the day and says gently, "My child, you are getting too fond of your pleasure, or your business, or your loved ones. Do you take your pleasure temperately, or do you like to see the wine-drop sparkle in the cup? If so, beware. Are you hasting to get rich? See that you are not with those who use false weights and balances, and be ye true and just in all your dealings." This illness, then, gives us time to think over our shortcomings, and to find out whether our motives are pure and leading us in the broad or in the narrow way.

Do we speak the truth even though it may lead us into difficulties, do we say our private prayers regularly, and do we worship in God's House, fine or wet, convenient or inconvenient? These are the points on which we should dwell, asking our dear Lord to give us His Holy Spirit to grow daily more like Him, for this may be a call to set our houses in order. If we obey it, happy will it be for us in the future. We will try and remember that we are not sent into this world for our own pleasure, but for the Almighty's, who would have us serve and please Him only, and the rest of our lives shall be devoted to His glory and praise.

### To Nurses.

If we but fix our thoughts above,
  And live our lives for God and man;
If all our motive power be love,
  And each one does the most she can,

We shall not simply live and fade
  Like earthly flowers that bloom and die,
For every noble deed is made
  To reach through all eternity.

So courage take and labour on,
  Spend and be spent, 'tis not in vain,
For though we often sow in tears,
  The richer harvests we shall gain!

NURSE BEATRICE FURNIVLE

